# Effects of six weeks outdoor *versus* treadmill running on physical fitness and body composition in recreationally active young males: a pilot study

**DOI:** 10.7717/peerj.13791

**Published:** 2022-07-27

**Authors:** Gaurav Singh, Gaurav Kushwah, Tanvi Singh, Rodrigo Ramírez-Campillo, Rohit K. Thapa

**Affiliations:** 1 School of Physical Education and Sports, Rashtriya Raksha University, Gandhinagar, India; 2 Exercise and Rehabilitation Sciences Laboratory, School of Physical Therapy, Faculty of Rehabilitation Sciences, Universidad Andres Bello, Santiago, Chile

**Keywords:** Exercise, Movement, Physical activity, Human physical conditioning, Musculoskeletal and neural physiological phenomena, Physical exertion, Muscle strength, High-intensity interval training, Athletic performance, Motor activity

## Abstract

**Background:**

Running as exercise may be performed either on an outdoor surface or treadmill surface. However, previous research has indicated that the nature of both the surfaces differ significantly and therefore the training outcomes from running in these surfaces may also vary.

**Aim:**

Therefore, the aim of this pilot study was to compare the effects of 6-weeks of supervised outdoor running (OT) *vs* treadmill running (TT) on physical fitness and body composition in recreationally active young males.

**Methods:**

Participants (age: 19.82 ± 1.28 years, height: 172.6 ± 4.9 cm, body mass: 64.3 ± 8.7 kg) were randomly assigned to OT (*n* = 14) or TT (*n* = 14), and assessed for physical fitness, *i.e*., 50 m sprint, cardiorespiratory endurance (*i.e*., 1,600 m run time-trial), standing long jump (SLJ), flexibility (*i.e*., sit-and-reach test), and upper-body muscle endurance (*i.e*., push-ups repetitions), alongside body composition, *i.e*., body mass, body mass index (BMI), fat percentage, fat free mass, and leg skeletal muscle mass (SMM). A two (pre-post intervention) by two (OT, TT) mixed ANOVA analysed exercise-specific effects. For significant group-by-time interactions, Bonferroni adjusted paired (within-group) and independent (between-group comparisons at post) t-tests were used for post-hoc analyses.

**Results:**

Significant time-effect was found in all physical fitness variables (all *p* < 0.001, η_p_^2^ = 0.67–0.91), body mass (*p* = 0.23, η_p_^2^ = 0.18), BMI (*p* = 0.009, η_p_^2^ = 0.24), body fat percentage (*p* = 0.001, η_p_^2^ = 0.38), and leg SMM (*p* = 0.002–0.007, η_p_^2^ = 0.25–0.33). Significant group-by-time interaction was found for 50 m sprint (*p* = < 0.001, η_p_^2^ = 0.74), 1,600 m run (*p* = 0.001, η_p_^2^ = 0.35), and SLJ (*p* < 0.001, η_p_^2^ = 0.43), favouring OT. Group-specific post-hoc tests showed improvements in all physical fitness variables after OT (*p* = < 0.001–0.001, *g* = 0.69–2.32, %Δ = 3.0–12.4) and TT (*p* = < 0.001–0.017, *g* = 0.15–0.65, %Δ = 0.9–11.7), and fat percentage after OT and TT (*p* = 0.002–0.041, g = 0.14–0.26, %Δ = 4.3–6.0). However, leg SMM decreased in TT (*p* = 0.001–0.004, g = 0.14–0.15, %Δ = 6.2–6.7).

**Conclusions:**

Both OT and TT improved physical fitness and decreased fat percentage. However, compared to TT, the OT intervention preserved leg SMM and induced greater physical fitness improvements.

## Introduction

Physical fitness and body composition are important health indicators for individuals (Chiriboga & Ockene, 2016; Myers et al., 2015; Ortega et al., 2018), and is shown to improve through physical activities, such as running (Chalfoun et al., 2016; Idrizovic et al., 2021). Indeed, running is also associated with improved cardiorespiratory fitness (Willoughby et al., 2016), muscular endurance (Menz et al., 2019), cardiovascular health (Chalfoun et al., 2016; Idrizovic et al., 2021), and mental well-being (Bowler et al., 2010; Thompson Coon et al., 2011). Furthermore, running also improves mood and helps in exercise adherence (Oswald et al., 2020). The beneficial effects of running may be attributed to its positive effects on the cardiovascular and respiratory system (Delgado-Floody et al., 2019).

Running exercise can be performed on different type of surfaces (*e.g*., outdoor track, treadmill), potentially affecting the outcomes of running exercise due to differences in the mechanical properties (Colino et al., 2020). For example, compared to outdoor sports surfaces (*e.g*., artificial turf, track), running on treadmills increase shock absorption and vertical deformation (Colino et al., 2020). Additionally, compared to treadmill running, increased averaged electromyography activity for lower limb muscles was found in grass (*i.e*., tibialis anterior, peroneus longus, soleus, gastrocnemius medialis, vastus medialis, and rectus femoris) and concrete (*i.e*., gastrocnemius lateralis, semitendinosus, gluteus maximus) (Yaserifar & Souza Oliveira, 2022). Similarly, Wank, Frick & Schmidtbleicher (1998) reported lower leg swing amplitude, vertical displacement, and vertical and horizontal velocity variance in treadmill running compared to synthetic surface running, resulting in lower external work because of lower lifting of the body mass per step and lower range in cyclic re-acceleration. Indeed, Morin & Sève (2011) reported differences in sprinting biomechanics between treadmill and over-ground sprinting, with sprint speed constantly changing throughout the duration of the over-ground sprint compared to treadmill sprinting. Moreover, among male recreational and competitive runners, higher scores of psychological pride, tension and effort were observed with outdoor compared to treadmill running (Kerr et al., 2006).

The aforementioned differences between running outdoor *vs* treadmill may be of significance for individuals targeting physical fitness and body composition improvements. Indeed, compared to control condition, Dorgo et al. (2020) reported improvements in sprint time, maximal treadmill speed, total body fat, and leg lean muscle mass after a 6-week sprint training programme in treadmill or over-ground surface, although with each type of intervention providing some specific adaptations (*e.g*., greater treadmill sprint performance after treadmill training; greater leg lean muscle mass after over-ground training). Additionally, Singh et al. (2022) reported higher session’s rating of perceived exertion during outdoor running compared to treadmill running sessions.

However, the literature on training effects of outdoor *vs* treadmill running on physical fitness and body composition among young recreationally active male individuals is scarce. Thus, the aim of this novel study was to compare the effects of 6-weeks supervised outdoor running *vs* treadmill running on measures of physical fitness and body composition in young recreationally active male adults. Considering that training on an outdoor surface may provide higher muscle activation, psychobiological stimulus, and biomechanical demands (Colino et al., 2020; Kerr et al., 2006; Wank, Frick & Schmidtbleicher, 1998; Yaserifar & Souza Oliveira, 2022) compared to a treadmill surface, we hypothesized larger improvements in both physical fitness and body composition outcome variables following 6 weeks of outdoor running.

## Materials and Methods

### Participants

To calculate the required sample size, a freeware statistical software tool (G*Power; University of Düsseldorf, Düsseldorf, Germany) was used. The following variables were included in the *a priori* power analysis: study design, two groups; test, retest; effect size of 0.66 based on a previous study that investigated the effects of training in unstable surface (*i.e*., similar to outdoor running) *vs* stable surface (*i.e*., similar to treadmill running) in adolescent male soccer players on countermovement jump (Granacher et al., 2015); alpha error < 0.05; nonsphericity correction = 1; correlation between repeated measures = 0.5; desired power (1-ß error) = 0.80.

**Figure 1:**
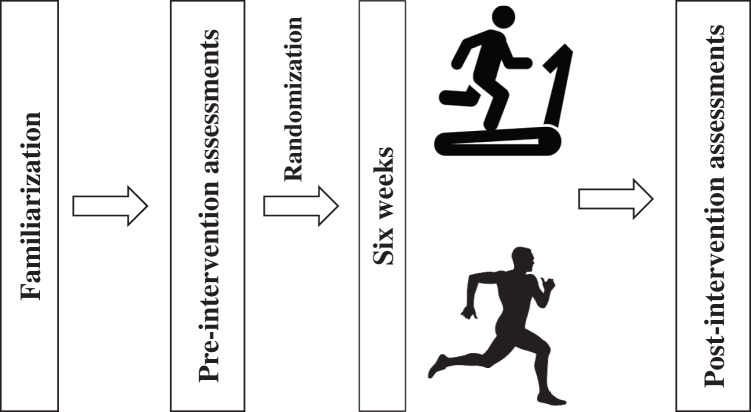
Schematic representation of study design.

The results of the *a priori* power analysis indicated that a minimum of eight participants would be needed for each group to achieve statistical significance for jump performance (*i.e*., standing long jump (SLJ)). Thereafter, 28 recreationally active (2–3 moderate-intensity exercise sessions per week; sporadic recreational sport participation) male participants were recruited for the study. A greater number of participants was recruited considering potential drop out due to injury (*i.e*., not associated to the intervention), lack of time, or related reasons. A graphical depiction of the study design is shown in Fig. 1. To be eligible, participants (i) had to be free of lower limbs injuries during the 6 months prior to the start of the study; (ii) had to participate in physical fitness training programs (*i.e*., three sessions per week). The participants were randomly assigned to an outdoor running group (OT; *n* = 14) or a treadmill running group (TT; *n* = 14) using an online randomization software (www.randomizer.org). The participants of each group possessed similar demographic characteristics (Table 1). Prior to the start of the study, all participants received information on potential risks and benefits of this study. Thereafter, informed consent was obtained. The local ethical committee of Rashtriya Raksha University approved this study (approval number: RRU/R&P/RRUEC/6^th^/187/2021).

**Table 1:** Demographics of the participants.

	Mean ± standard deviation		
	Outdoor running group (*n* = 14)	Treadmill running group (*n* = 14)	*p-*value
Age (years)	20.2 ± 0.9	19.4 ± 1.5	0.105
Height (cm)	171.4 ± 4.1	173.9 ± 5.6	0.185
Body mass (kg)	64.5 ± 7.0	64.2 ± 10.3	0.916

### Procedure

One week before baseline assessment, the participants performed two familiarization sessions for the testing procedures. Demographic data were collected during the familiarization sessions. Participants were asked to refrain from any strenuous activity for 24 h and eating for three hours prior to testing. A two × two within-subject and between-subject, randomized design was used to compare the effect of training intervention (*i.e*., OT, TT) on physical fitness (*i.e*., 50 m linear sprint, 1,600 m run, SLJ, flexibility, push-up) and body composition variables (*i.e*., body mass, body mass index (BMI), fat percentage, fat free mass, segmental muscle mass (SMM) of legs). In addition, the participants were required to maintain their nutritional habits, and this was requested repeatedly during intervention training sessions. Measurements were performed during similar hours before and after the intervention, across two consecutive days, with 50 m sprint, 1,600 m run, SLJ, flexibility and push-up recorded on day one and body composition variables recorded on day two. The sequence of test order was similar for all the participants. Upon arrival for testing on days one participants underwent 10-min general warm-up procedure. For the outdoor assessments the temperature (31–34 °C), humidity (26–29%), and wind velocity (7 km.h^−1^) were similar before and after the intervention.

#### Training intervention

During the 6-weeks intervention, both groups completed 18 running sessions (*i.e*., three sessions/week) in either treadmill (*i.e*., model S-TRC; Star Trac, Irvine, CA, USA) or outdoor surface (*i.e*., 400-m athletic cinder track). Each training sessions were approximately 50–60 min in duration *i.e*., 10–15 min of warm-up (*e.g*., jogging progressing to short sprints, dynamic stretching exercises), 30 min of running, and 10–15 min of warm-down. A minimum of 48 h of inter-session recovery was allowed between running sessions. Both groups were continuously supervised and matched for training intensity (*i.e*., 60–80% of predicted maximal heart rate) during the running sessions using heart rate monitors placed on the chest using a strap (Polar H10, Kempele, Finland). A coach was assigned to supervise the heart rate using the Polar Beat application (version 3.5.5) and provided continuous verbal feedback to the participants. The speed of the treadmill was adjusted (TT group) or the participants were asked to increase/decrease their pace (OT group) in order to match the required training intensity. Training volume (total minutes) was similar between the exercise groups (Table 2). Additionally, both OT and TT underwent a volume-matched (total repetitions) circuit strength training session once per week, including exercises requiring participants own body mass as resistance.

**Table 2:** Training protocol included in the study.

	Outdoor running group	Treadmill running group
Monday, Wednesday, and Friday	30 min running*Intensity: 60–80% of predicted maximal heart rate	30 min running*Intensity: 60–80% of predicted maximal heart rate
Saturday	Body weight strength training**	Body weight strength training**

**Notes:**

* Running sessions included (i) warm-up of 10–15 min duration which included sprints and dynamic stretching exercise (ii) post running session warm-down of 10–15 min duration which included walking and static stretching exercises.

** Participants performed exercises such as push-ups, planks, squats, jump squats, lunges, and crunches in circuit format.

### Assessments

#### 50-m linear sprint

Participants were instructed to stand behind a marked line and start only after the sound of the clapper. Two independent coaches who were not part of this study were recruited as timekeepers (between timekeepers interclass correlation coefficient (ICC) = 0.98) and assigned to record the timing of each trial using a hand stopwatch (Casio S053 HF-70W-1DF; Casio Computer Co., Ltd., Tokyo, Japan). The assessors were unaware of the group allocated to the participants. The average of time recorded by both timekeepers was used for inclusion. Three trials were conducted with a recovery period of approximately one minute between each trial, and the fastest trial was selected for analysis. The ICC for test-retest was 0.94.

#### 1,600 m run

The test was conducted in a standard 400 m track. Participants were instructed to run 1,600 m in shortest time possible. Standing start was adopted for the test. Two independent coaches who were not part of this study were recruited as timekeepers (between timekeepers ICC = 0.97) and assigned to record the timing using a hand stopwatch (Casio S053 HF-70W-1DF; Casio Computer Co., Ltd., Tokyo, Japan). The assessors were unaware of the group allocated to the participants. The average of time recorded by both timekeepers was used for inclusion. Only one maximal valid trial was allowed.

#### Standing long jump

The SLJ was conducted on an outdoor track. Participants were instructed to stand behind a marked line with feet slightly apart, and to take off with both legs and land on both legs. Arm-swing and countermovement were allowed. A verbal encouragement to jump as far as possible was provided by the independent assessors who were blinded to group allocation. The measurement was recorded from the take-off line to the nearest point of contact on the landing (*i.e*., back of the heels). Three jumps were recorded with ~1 min of between-jumps recovery. The longest jump was used for the analysis. The ICC for test-retest was 0.72.

#### Flexibility

To measure flexibility, the sit-and-reach test was applied, using a testing box (12-1085 Baseline sit and reach trunk flexibility box, Baseline Measurement Instruments; Fabrication Enterprises INC, Elmsford, NY, USA). The protocol for the test has been reported elsewhere (Ramírez-Campillo et al., 2015). Three trials were conducted with an inter-trial recovery of ~1 min. The best score was selected for the analysis. The ICC for test-retest was 0.77.

#### Push-ups

To measure upper-body muscular endurance the maximal number of push-ups performed were counted. The protocol for push-ups has been reported elsewhere (McManis, Baumgartner & Wuest, 2000). Only one maximal valid trial was allowed.

#### Body composition

Body mass, body fat percentage, fat free mass, and leg SMM were measured with bioelectrical impedance technology (BC300 whole-body and segmental Body Composition Analyzer; ACCUNIQ BC300, SELVAS Healthcare, Seoul, Republic of Korea). The participants wore minimal clothes and removed all metallic belongings. Thereafter, the participants stood up in the measurement equipment in an anatomical position with their arms open and away from their body at about a 30° angle; such position was maintained until the end of the measurement, while maintaining a quiet stance. The BMI was calculated as body mass (kg) divided by height (m) squared. The assessment was conducted in the morning hours and participants were requested to avoid any food or water intake in the three hours preceding the measurements.

### Statistical analysis

The analyses were conducted using IBM SPSS version 20.0.0 (IBM, Armonk, NY, USA). Data normality was verified using the Shapiro-Wilk test. Data are presented as means and standard deviations. A two (pre-post intervention) by two (OT, TT) mixed ANOVA was used to analyse the exercise-specific effects. In addition, in case of significant group-by-time interactions, Bonferroni adjusted paired (within-group) and independent (between-group comparisons at post) t-tests were used for post-hoc analyses. Percentage change scores were also calculated for each variable in each group using the equation in Microsoft excel sheet: [(mean_post_ − mean_pre_)/mean_pre_] × 100. Effects sizes (ES) in the form of partial eta squared (η_p_^2^) were used from ANOVA output. Hedge’s *g* derived from paired t-test and independent t-test was calculated to assess changes between pre-post measurements testing for each group as well as between group differences at post measurements. The magnitude of effects for η_p_^2^ was interpreted as small (<0.06), moderate (≥0.06–0.13), and large (≥0.14) (Cohen, 1988), while Hedge’s *g* was interpreted as trivial (<0.2), small (0.2–0.6), moderate (>0.6–1.2), large (>1.2–2.0), very large (>2.0–4.0) and extremely large (>4.0) (Hopkins et al., 2009). The ICC between trials was interpreted as poor (<0.5), moderate (0.5–0.75), good (0.75–0.9), and excellent (>0.9) reliability based on the lower bound of the 95% confidence interval (CI; ICC_95%CI_ _lower bound_) (Koo & Li, 2016). Statistical significance was set at *p* ≤ 0.05.

## Results

The participants in OT (*n* = 14) and TT (*n* = 14) were similar in age, height and body mass during group allocation (*p* = 0.105–0.916) (Table 1). There were no injuries reported by the participants during any of the intervention. In addition, all participants in both groups adhered to the training protocol and attended all allocated sessions (*i.e*., 18 running sessions). There was no drop out reported during the study.

The results for all dependent variables are presented in Table 3, with a graphical representation of pre-post percentage change in Fig. 2. No baseline differences (independent t-test p = 0.06–0.916) were observed between OT and TT groups in any of the dependent variables.

**Table 3:** Statistical comparisons for changes in physical fitness and body composition variables in outdoor and treadmill running group.

	Variable	Outdoor running group (*n* = 14)			Treadmill running group (*n* = 14)			Main effect (time)	Time × group
		**Pre**	**Post**	***p*-value [g]** **Magnitude**	**Pre**	**Post**	***p*-value [g]** **Magnitude**	***p*-value [η**_**p**_^**2**^ **]** **Magnitude**	***p*-value [η**_**p**_^**2**^ **]** **Magnitude**
		**Mean ± standard deviation**			**Mean ± standard deviation**				
Physical #?>fitness	50 m sprint (s)	7.57 ± 0.31	7.34 ± 0.31	<0.001*^#^ [0.72]Moderate	7.74 ± 0.44	7.67 ± 0.44	<0.001*^#^ [0.15]Trivial	<0.001 [0.91]Large	<0.001 [0.74]Large
	1,600 m run (min)	7.71 ± 0.37	7.07 ± 0.46	<0.001* [1.49]Large	7.40 ± 0.43	7.19 ± 0.50	0.017* [0.44]Small	<0.001 [0.67]Large	0.001 [0.35]Large
	Standing long jump (m)	1.93 ± 0.11	2.17 ± 0.09	<0.001*^#^ [2.32]Large	1.95 ± 0.16	2.06 ± 0.17	<0.001*^#^ [0.65]Moderate	<0.001 [0.87]Large	<0.001 [0.43]Large
	Flexibility (cm)	22.64 ± 2.68	24.57 ± 2.77	0.001* [0.69]Moderate	22.36 ± 3.65	23.79 ± 3.21	0.009* [0.40]Small	<0.001 [0.46]Large	0.494 [0.18]Large
	Push-up (repetitions)	21.00 ± 3.70	23.50 ± 2.93	<0.001* [0.73]Moderate	19.64 ± 3.71	21.93 ± 3.93	<0.001* [0.58]Moderate	<0.001 [0.71]Large	0.726 [0.01]Small
Body composition	Body mass (kg)	64.51 ± 7.00	63.96 ± 6.16	0.167 [0.08]Trivial	64.15 ± 10.33	63.39 ± 9.14	0.56 [0.08]Trivial	0.023^#^ [0.18]Large	0.685 [0.01]Small
	Body mass #?>index (kg.m^−2^)	21.96 ± 2.14	21.77 ± 1.92	0.118 [0.09]Trivial	21.19 ± 2.76	20.90 ± 2.44	0.24 [0.11]Trivial	0.009^#^ [0.24]Large	0.586 [0.01]Small
	Body fat (%)	19.51 ± 4.94	18.33 ± 3.94	0.002* [0.26]Small	17.36 ± 5.74	16.61 ± 4.86	0.041* [0.14]Trivial	0.001^#^ [0.38]Large	0.373 [0.03]Small
	Fat free mass (kg)	51.68 ± 3.79	51.46 ± 3.89	0.304 [0.06]Trivial	52.66 ± 5.64	52.38 ± 5.34	0.199 [0.05]Trivial	0.106 [0.10]Moderate	0.850 [0.01]Small
	SMM right leg (kg)	2.30 ± 0.77	2.24 ± 0.74	0.173 [0.08]Trivial	2.08 ± 1.00	1.94 ± 0.84	0.001* [0.15]Trivial	0.002^#^ [0.33]Large	0.131 [0.09]Moderate
	SMM left leg (kg)	2.28 ± 0.76	2.24 ± 0.74	0.331 [0.05]Trivial	2.09 ± 1.00	1.96 ± 0.84	0.004* [0.14]Trivial	0.007^#^ [0.25]Large	0.141 [0.08]Moderate

**Notes:**

*g*, Hedges *g* effect size; η_p_^2^, partial eta squared; SMM, segmental muscle mass.

* Significant difference between pre- and post-intervention measures.

# Significant difference at post-intervention measures between groups.

**Figure 2:**
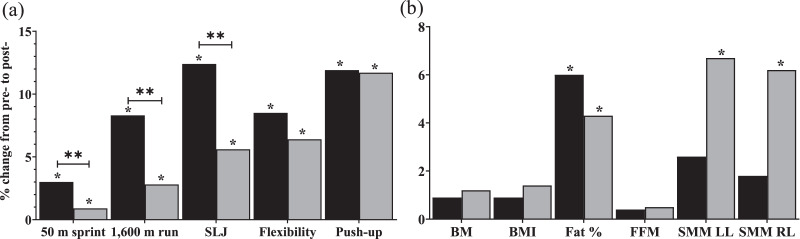
Relative (%) change in (A) physical fitness variables and (B) body composition variables between before (pre) and after (post) training intervention for the outdoor (OT; black bars) and treadmill (TT; grey bars) running groups. Note: BM, body mass; BMI, body mass index; FFM, fat free mass; LL, left leg; RL, right leg; SLJ, standing long jump; SMM, skeletal muscle mass; *, significant difference from pre- to post; **, significant group-by-time interaction.

## Physical fitness variables

There was significant main effect of time on 50 m sprint (*p* < 0.001, η_p_^2^ = 0.91), 1,600 m run (*p* < 0.001, η_p_^2^ = 0.67), SLJ (*p* < 0.001, η_p_^2^ = 0.87), flexibility (*p* < 0.001, η_p_^2^ = 0.46) and push-up (*p* < 0.001, η_p_^2^ = 0.71). However, significant time × group effect was observed in 50 m sprint (*p* < 0.001, η_p_^2^ = 0.74), 1,600 m run (*p* = 0.001, η_p_^2^ = 0.35) and SLJ (*p* < 0.001, η_p_^2^ = 0.43). Furthermore, both OT and TT improved physical fitness of the participants, however, OT showed greater improvement in 50 m sprint (*p* < 0.001, *g* = 0.91, %Δ = 3.0 **vs* p* < 0.001, *g* = 0.15, %Δ = 0.9), 1,600 m run (*p* < 0.001, *g* = 1.49, %Δ = 8.3 **vs* p* = 0.017, *g* = 0.44, %Δ = 2.8), SLJ (*p* < 0.001, *g* = 2.32, %Δ = 12.4 **vs* p* <0.001, *g* = 0.65, %Δ = 5.6), and flexibility (*p* = 0.001, *g* = 0.69, %Δ = 8.5 **vs* p* = 0.009, *g* = 0.40, %Δ = 6.4). Similar improvements were observed in push-ups (*p* < 0.001, *g* = 0.73, %Δ = 11.9 **vs* p* < 0.001, *g* = 0.58, %Δ = 11.7). Between-group difference at post-test were observed in 50 m sprint (*p* = 0.029, *g* = 0.84) and SLJ (*p* = 0.05, *g* = 0.79).

## Body composition variables

There was significant main effect of time on body mass (*p* = 0.023, η_p_^2^ = 0.18), BMI (*p* = 0.009, η_p_^2^ = 0.24), body fat percentage (*p* = 0.001, η_p_^2^ = 0.38), SMM right leg (*p* = 0.002, η_p_^2^ = 0.33), and SMM left leg (*p* = 0.007, η_p_^2^ = 0.25), but not on fat free mass (*p* = 0.106, η_p_^2^ = 0.10). In addition, no group × time effect was observed for any variables (*p* = 0.131–0.850, η_p_^2^ = 0.01–0.09). Furthermore, both OT and TT showed significant improvement in body fat percentage with slightly greater improvement in OT (*p* = 0.002, *g* = 0.26, %Δ = 6.0 **vs* p* = 0.041, *g* = 0.14, %Δ = 4.3). However, leg SMM was significantly decreased following TT but not OT (*p* = 0.001–0.004, *g* = 0.14–0.15, %Δ = 6.2–67 **vs* p* = 0.173–0.331, *g* = 0.05–0.08, %Δ = 1.8–2.6). No between-group difference at post-test were observed in any variables.

## Discussion

Our novel findings revealed that both OT and TT improved physical fitness and body composition, although greater improvements were noted in 50 m sprint, 1,600 m run and SLJ following 6-weeks of OT. Moreover, greater magnitude of improvement was noted after OT compared to TT in flexibility, and body fat reduction. Further, a reduction of leg SMM was noted after TT, while OT preserved leg SMM.

### Physical fitness variables

The physical fitness variables were significantly improved in both the running groups. These improvements may be attributed to myriad of factors associated with running. For example, improvement in 50 m sprint and 1,600 m performance may be associated with better running economy (Barnes & Kilding, 2015). Running endurance training can lead to a wide range of cardiorespiratory, metabolic, physiological, biomechanical or neuromuscular adaptations (Barnes & Kilding, 2015). Indeed, endurance training has shown to increase the morphology and functionality of skeletal muscle mitochondria (Holloszy et al., 1977). Furthermore, training induced changes may also have occurred such as improved skeletal muscle buffer capacity (Gore et al., 2001) and hematological changes (*e.g*., increased red blood cells) (Burtscher et al., 1996). In addition, we also observed improvement in SLJ performance. This improvement may be particularly attributed to enhanced stretch-shortening cycle function of the lower limbs following running intervention. Furthermore, we observed improvement in flexibility in both running groups. This improvement in flexibility may have occurred due to increased muscle and collagen strength of lower limbs, tensile strength of tendons and ligaments, and muscle contractility (Fatouros et al., 2002). Moreover, in our study the participants also performed static stretching following running sessions (*i.e*., during warming-down), which may also be responsible for improvement in flexibility (Hadjicharalambous, 2016). In addition, both running groups also underwent a supplementary strength training session (*i.e*., circuit training format) one session/week, involving push-ups and planks, which may explain the similar improvements in the push-up test in both running groups.

However, we observed a greater improvement in 50 m sprint, 1,600 m run, and SLJ after OT compared to TT. One possible mechanism for these findings may be the greater neuromuscular activation that occurred during OT compared to TT, contributing to more enhanced functional and neural adaptations. Indeed, Yaserifar & Souza Oliveira (2022) reported increased lower limbs muscle activity when running on grass (*i.e*., tibialis anterior, peroneus longus, soleus, gastrocnemius medialis, vastus medialis, and rectus femoris) and concrete (*i.e*., gastrocnemius lateralis, semitendinosus, gluteus maximus) compared to treadmill running. Moreover, lower leg swing amplitude, vertical displacement, and vertical and horizontal velocity variance was noted in treadmill running compared to synthetic surface running, leading to lower external work in treadmill running because of lower lifting of the body mass per step and lower range in cyclic re-acceleration (Wank, Frick & Schmidtbleicher, 1998). The greater intensity of OT compared to TT might have induced greater muscle recruitment, motor unit firing rate and synchronization in the timing of neural discharge during high intensity muscular contraction (Aagaard et al., 2002; Sale, 1987; Semmler et al., 2004), thus greater improvements in 50 m sprint, 1,600 m run, and SLJ. Similarly, the greater flexibility improvements after OT compared to TT (moderate *vs* small) may be attributed to physiological adaptations (*e.g*., larger increase in muscle and collagen strength). Nonetheless, as no physiological data was collected, any mechanistic explanation remain speculative.

### Body composition variables

Body fat percentage was reduced in both OT and TT. Similar findings were observed in a study by Dorgo et al. (2020), who compared sprint training on treadmill *vs* over-ground surface. The finding in our study may be attributed to the aerobic nature of the running intervention, as a previous study reported aerobic training as a key contributor to fat loss (Ismail et al., 2012). Indeed, Hottenrott, Ludyga & Schulze (2012) reported decrease in fat percentage following a 12-weeks endurance running intervention in recreationally active runners. Similarly, Milanović et al. (2015) reported decrease in fat percentage following a 12-weeks running intervention program in healthy untrained males. However, greater fat percentage reduction was observed in OT compared to TT (small *vs* trivial). Compared to running on a treadmill surface, running on outdoor surfaces (*e.g*., grass, concrete) increase lower limbs muscle activity (Yaserifar & Souza Oliveira, 2022), vertical displacement, and vertical and horizontal velocity variance, leading to greater external work because of higher lifting of the body mass per step and greater range in cyclic re-acceleration (Wank, Frick & Schmidtbleicher, 1998). Therefore, OT may have induced greater total energy expenditure (*i.e*., negative energy balance), the main factor for fat loss (Slentz et al., 2004). It is then probable that OT may increase total energy expenditure in young males compared to TT, resulting in greater reduction of body fat percentage. In addition, we observed trivial negative changes (*i.e*., decrease) in SMM of both legs in TT but not in OT. This finding suggests that OT preserved the SMM while decreasing the body fat percentage. The possible mechanistic reason for this might be the higher neuromuscular activation achieved during running in outdoor surface (Yaserifar & Souza Oliveira, 2022), which may have induced sufficient adaptions required to preserve the SMM. Therefore, OT may be suggested to young individuals with the goal of body fat reduction while preserving SMM of leg. In addition, we observed no changes in body mass, BMI, or fat free mass in either running group. However, there was a large magnitude main effect of time on the body mass and BMI. Running as an intervention seems to be an effective strategy in reducing body mass and improve BMI.

Of note, the participants included in our study were recreationally active adult males. This population is usually characterized for a meaningful window of adaptation to training intervention, particularly neuromuscular adaptations (Sale, 1987). Indeed, running intervention may increase activation of prime movers during the running action, and improved changes in the activation of synergist and antagonists muscles (Sale, 1987), thereby increasing efficacy and delaying fatigue of the contractile mechanism (Tamaki et al., 1998). Since significant neuro-mechanical differences have been reported while running in different surfaces (Colino et al., 2020; Dorgo et al., 2020; Kerr et al., 2006; Singh et al., 2022; Wank, Frick & Schmidtbleicher, 1998; Yaserifar & Souza Oliveira, 2022), it would be interesting to hypothesise in future studies that differences observed in outcome measures between OT and TT (*i.e*., recreationally-active participants) in our study is related to initial neural adaptations. However, how high-level athletes (*e.g*., endurance runners) would respond to OT and TT is yet unknown. Indeed, previous studies which compared the differences between treadmill and outdoor running included only recreationally active participants (*e.g*., recreationally active men, physical education students, physically active males, recreational runners, college-age male students) (Dorgo et al., 2020; Kerr et al., 2006; Singh et al., 2022; Wank, Frick & Schmidtbleicher, 1998; Yaserifar & Souza Oliveira, 2022). Therefore, the findings of this study should not be extrapolated to high-level athletes.

### Limitations

There are some limitations of our study which should be acknowledged. First, the participants involved in this study were recreationally active male individuals. Therefore, the validity of current findings in other populations (*e.g*., highly trained runners; females) needs future confirmation. Second, the training intervention was limited to 6-weeks of duration. Although significant changes for both running groups were noted after 6-weeks, a longer duration study may be needed to determine the long-term trend of adaptation after OT and TT for the different outcomes included in this study. Indeed, as a pilot study, our research was designed as proof-of-concept of the OT *vs* TT approach, and although we conducted a sample size estimation for two independent training groups, larger sample sizes, including a controlled design, may be required to sustain current findings. Indeed, as other pilot studies (Lichtenstein et al., 2020), current findings (*e.g*., effect sizes) serves as a basis for a long-term intervention. Third, future studies may clarify if the bodyweight strength training session, included in both intervention groups, affected current findings. Fourth, the absence of biomechanical or physiological comparisons in our study. Such comparison may provide better interpretation of the results. Finally, although we request the participants to maintain their habitual nutritional habits during the intervention, no formal measurement was performed, potentially affecting body composition measurements.

## Conclusions

Both OT and TT improved physical fitness and decreased body fat percentage after 6-weeks in young males. However, OT induced greater improvements in 50 m sprint, 1,600 m run, SLJ, preserved leg SMM, and induced greater magnitude of body fat percentage reduction compared to TT. Therefore, if the goal of a young male is to improve physical fitness and body composition, OT seems a safe and effective exercise training strategy, with the added benefit of requiring to access special equipment (*e.g*., treadmill). Nonetheless, practitioners may opt either OT or TT based on outdoor environmental conditions, availability of treadmills, and individual preferences, need analysis, and goals.

## Supplemental Information

10.7717/peerj.13791/supp-1 Supplemental Information 1 Raw data for descriptive variables and dependent variables.

## Additional Information and Declarations

### Data Availability

The following information was supplied regarding data availability: The raw measurements are available in the Supplemental Files.
